# AF1q Expression Associates with CD44 and STAT3 and Impairs Overall Survival in Adenoid Cystic Carcinoma of the Head and Neck

**DOI:** 10.1007/s12253-019-00696-z

**Published:** 2019-07-04

**Authors:** Lorenz C. Kadletz, Faris F. Brkic, Bernhard J. Jank, Sven Schneider, Julia Cede, Rudolf Seemann, Elisabeth S. Gruber, Elisabeth Gurnhofer, Gregor Heiduschka, Lukas Kenner

**Affiliations:** 1 Department of Otorhinolaryngology and Head and Neck Surgery, Medical University of Vienna, Vienna, Austria; 2 Department of Craniomaxillo and Facial Surgery, Medical University of Vienna, Vienna, Austria; 3 Department of General Surgery, Division of Surgery, Comprehensive Cancer Center, Medical University of Vienna, Vienna, Austria; 4 Department of Experimental Pathology and Laboratory Animal Pathology, Medical University of Vienna, Vienna, Austria; 5 Ludwig Boltzmann Institute for Cancer Research, Vienna, Austria; 6 Department of Experimental Pathology and Laboratory Animal Pathology, University of Veterinary Medicine, Vienna, Austria

**Keywords:** Adenoid cystic carcinoma, AF1q, Wnt, STAT3, Prognosis

## Abstract

Salivary gland malignancies of the head and neck form a heterogeneous group. Adenoid cystic carcinomas are an aggressive entity of salivary gland malignancies characterized by frequent distant metastases and poor response to radio- and chemotherapy. AF1Q is a MLL fusion partner, which can activate Wnt and STAT3 signaling. Recently, overexpression of AF1q has been identified as a poor prognosticator in patients of different malignancies. A total of 46 patients with adenoid cystic carcinoma were immunohistochemically evaluated for expression of AF1q and clinical outcome was analyzed in this context. Additionally, STAT3 and the Wnt downstream target CD44 were investigated and correlated with AF1q. AF1q was overexpressed in 52.2%. Overexpression of AF1q was associated with poorer overall survival (*p* = 0.03). Additionally, lymph node metastases and solid tumor parts were more frequently observed in AF1qhigh patients (*p* = 0.07 and 0.05, respectively). AF1q did not influence the occurrence of distant metastases. Expression of AF1q was associated with higher levels of STAT3 and CD44 (*p* = 0.003 and 0.006, respectively). AF1q is a novel prognostic marker for poor overall survival in adenoid cystic carcinoma patients. The deleterious effects on survival may be a result of promotion of the STAT3 and Wnt pathway.

## Introduction

Malignancies originating from salivary gland tissue are rare and have a reported incidence rate of 5–10 per 1,000,000. [1] In addition, salivary gland malignancies appear as a heterogeneous group with several different histologic subtypes. Currently, the WHO differentiates between 22 malignant epithelial tumors of the salivary glands. [2] Both their scarcity and heterogeneity make it particularly difficult to study and understand all histologic subtypes.

Adenoid cystic carcinoma (ACC) confronts patients and clinicians with several serious issues and aggressive features that were first described by Dockerty and Mayo in 1942. [3] This kind of salivary gland carcinoma is characterized by distant metastases, high rate of recurrences and perineural invasion. Overall survival rates are reported to approximate 80% after 5 years and 60% after 10 years. [4] Nevertheless, over 40% of all ACC patients are confronted with distant metastases during the course of their disease. [5] Particularly patients diagnosed with the solid form of ACC are at a high risk of increased mortality and of developing distant metastasis. [6]

Park et al. have recently identified AF1q as a factor that promotes distant metastasis in breast cancer. [7] AF1q, a MLL fusion partner, was first described by Tse and colleagues as a fusion gene (1; 11) (q21; q23) that is specifically expressed in leukemic and hematologic precursor cells. [8] Although the chromosomal abnormality that was detected then is not observed in solid tumors, AF1q expression has been found in various other malignancies including solid tumors. [9, 10] The oncogenic potential of AF1q may be a result of its capability to activate the Wnt pathway. [7] AF1q and its cofactors TCF1, beta-catenin and LEF1 form a transcriptional complex and activate the expression of CD44, a downstream target of the Wnt pathway. Aberrant Wnt signaling is associated with invasive characteristics of ACC in-vitro. [11] Activation of the Wnt pathway through beta catenin is associated with decreased survival rates in patients with major and minor salivary gland malignancies. [12, 13] Additionally, expression of AF1q enhances the oncogenic STAT3 signaling pathway via upregulation of PDGF-B and consecutive phosphorylation of the PDGF receptor beta. [14] Inhibition of STAT3 may be therefore a promising strategy in the treatment of ACC. Specifically, Bu and colleagues have demonstrated that selective inhibition of STAT3 by the small molecule inhibitor S3I-201 results in decreased cellular proliferation, migration and invasion of ACC cell lines. [15]

In this context, we hypothesized that AF1q might be expressed in ACC of the head and neck. Moreover, we aimed to investigate its effects on patient survival and potential associations with the Wnt and STAT3 signaling pathways.

## Materials and Methods

### Patients

In this study patients with ACC that were operated on for ACC of major and minor salivary glands of the head and neck region at the Medical University of Vienna between 1996 and 2016 were included. Informed consent is obtained from every patient treated at the Vienna General Hospital that data may be used for studies. Inclusion criteria were met if the patient was diagnosed with ACC for the first time and aged over 18 years. Tracheal ACC were excluded from this study. TNM staging were assessed. Histologic reports were evaluated for grading according to Perzin/Szanto and Spiro depending on the predominant growth type (tubular, cribriform or solid) and for the presence of perineural and lymphovascular invasion. [16–18] Medical records including, especially time of initial diagnosis, last follow-up, death and local or distant recurrence were noted for survival time analysis. In addition, we assessed therapy at the time of initial diagnosis.

### Ethical Considerations

This study was approved by the institutional research board of the Medical University of Vienna (ECS 1517/2018). All methods were performed in accordance with the Good Clinical Practice guidelines and the Declaration of Helsinki.

### Tissue Micro-Array and Immunohistochemistry

Samples were collected from previously evaluated paraffin-embedded ACC specimens that were obtained through surgical resection or biopsies taken prior to initial therapy via a Galileo Tissue Micro-Array (TMA) CK Series-HTS Tissue computer assisted Microarray Platform (Integrated Systems Engineering Srl, Milan, Italy).

Consecutively, 4 μm sections were prepared and processed for immunohistochemical analysis.

Immunohistochemical stainings as well as histopathological analysis was performed by L.K. Immunohistochemical scores were calculated by adding up intensity (0, 1, 2 or 3) and percentage of positive cells (0–5% = 0, 5–40% = 1, 40–80% = 2 or > 80% = 3).

### Statistical Analysis

Rates of overall survival (OS) and disease-free survival (DFS) were calculated by means of the Kaplan-Meier method. Statistical differences between groups were compared using the log-rank test (Mantel-Cox). Hazard ratio, standard error of the mean and confidence intervals were calculated. Univariate and multivariate analysis using a Cox regression model were performed additionally.

Categorical variables were compared using Fisher’s exact test or Chi^2^ test. Mann-Whitney test was used for comparison of unpaired groups in case of ordinal data. In addition, ROC curves were created to calculate the Spearman correlation value r. A *p* value <0.05 was considered as statistically significant.

SPSS software (Version 21.0; SPSS, Inc., Chicago, IL, USA) and Prism GraphPad software (GraphPad Software, Inc., La Jolla, CA, USA) were used to analyze and graphically present the data.

## Results

### Patient Characteristics

A total of 86 patients were diagnosed with ACC of the head and neck and treated at the Medical University of Vienna in the period between 1996 and 2016. Out of these, 46 patients had adequate medical records and quantities of material for immunohistochemical analysis. A total of 13 patients had small primary tumors (T1 *n* = 6 and T2 *n* = 7). (Table 1) Most of our patients had locally advanced disease. In 8 patients the tumor was classified as T3, and in the majority of all patients (*n* = 25) a T4 tumor was present at initial diagnosis. Lymph node metastases were detectable in 10 patients and distant metastases in 3 patients at the time of initial diagnosis. High-grade tumors according to Perzin and Szanto were diagnosed in 9 patients. Five patients had more than 50% solid components and were therefore classified as high-grade according to the Spiro grading system. A total of 23 patients showed signs of perineural invasion, and lymphovascular invasion was microscopically detectable in 6 patients. During follow-up 25 patients died and 23 patients suffered from recurrent disease. In 18 patients distant metastases were found during their course of disease. Furthermore, 7 ACC patients were diagnosed with multiple recurrences during their course of disease. The vast majority of all patients (*n* = 43) was initially treated with surgical resection. Adjuvant therapy was applied in 20 patients.

**Table 1:** Basic data and descriptive statistics of patients with adenoid cystic carcinoma included in this study

	Number of patients	Percentage
Sex		
Male	20	43.5%
Female	26	56.5%
T-classification		
T1	6	13.0%
T2	7	15.2%
T3	8	17.4%
T4	25	54.4%
N-classification		
N+	10	21.7%
N0	36	78.3%
M-classification		
M0	43	93.5%
M1	3	6.5%
Perineural Invasion		
Yes	23	50.0%
No	23	50.0%
Lymphovascular Invasion		
Yes	6	13.0%
No	40	87.0%
Survival		
Dead	25	54.3%
Alive	21	45.7%
Recurrence		
Yes	23	50.0%
No	23	50.0%
Multiple recurrences		
Yes	7	15.2%
No	39	84.8%
Treatment		
Primary radiotherapy	2	4.3%
Initial chemotherapy	1	2.2%
Surgery	43	93.5%
Postoperative radiochemotherapy	10/43	23.3%
Postoperative radiotherapy	10/43	23.3%
Perzin and Szanto Grading		
1	12	26.1%
2	25	54.3%
3	9	16.6%
Spiro Grading		
1	29	63.1%
2	11	23.9%
3	5	10.9%
Histologic components		
Solid	4	8.7%
Tubular	3	6.5%
Cribriform	21	46.7%
Solid&Tubular	1	2.2%
Solid&Cribriform	6	13.0%
Tubular&Cribriform	9	19.6%
All three types	2	4.3%
Localization		
Parotid Gland	6	13.0%
Submandibular Gland	8	17.4%
Sublingual Gland	2	4.3%
Minor Salivary Gland	30	65.2%

### Expression of AF1q and its Impact on Survival

**Fig. 1:**
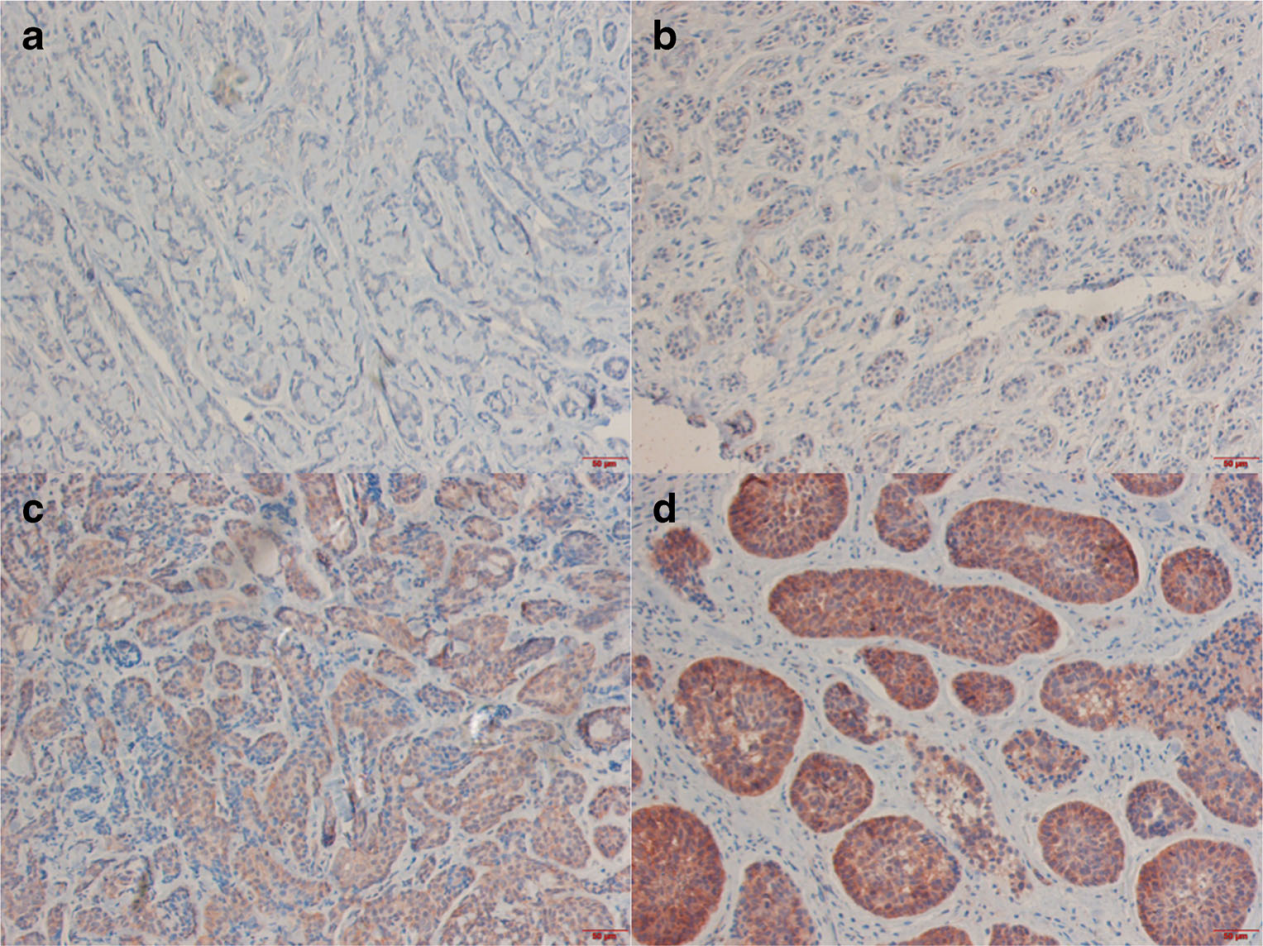
Samples of patients with adenoid cystic carcinoma (ACC) and predominately tubular growth pattern are shown in (**a**) and (**b**). In (**a**) no expression of AF1q could be observed and in (**b**) very low intensity could be observed in <5% of all tumor cells. Sample (**c**) shows a mixed growth pattern with moderate expression of AF1q. High expression of a solid typ is depicted in sample (**d**)

Out of 46 samples, AF1q expression was detected in 30 specimens, while 16 samples showed no signs of AF1q expression (Fig. 1a). Among the AF1q positive tumors, scores were distributed as follows: 6 patients had score 1–2 (low), 16 patients score 3–4 (moderate), and 8 patients score 5–6 (high) (Fig. 1b, c and d). To gain more power for survival analysis patients were stratified into two groups, AF1q low- (score = 0–2) and high-expressing (score = 3–6) tumors. Thus, a total of 22 patients were classified as AF1q^low^ and 24 patients as AF1q^high^.

First of all, we investigated the effects of AF1q in the context of several clinicopathologic parameters. As shown in Table 2, T1–4 tumors were equally distributed between both groups. Lymph node metastases were more often detected in patients with high expression of AF1q in primary tumors (9.1% vs. AF1q^high^ 33.3%). However, results were not statistically significant (*p* = 0.07). As AF1q promotes the formation of distant metastases in breast cancer patients [7], we compared the incidence of distant metastases at initial diagnosis and distant recurrences in dependence of AF1q expression. Surprisingly, we did not find any differences in the occurrence of distant metastases at different time points (*p >* 0.99 and *p =* 0.77 for initial diagnosis and distant recurrence, respectively). Solid components of ACC are usually associated with poor prognosis. In our study, solid parts were > 3 times more often observed in AF1q^high^ tumors (41.7% vs. 13.6%). Nevertheless, the level of significance was not reached (*p* = 0.051). Although solid components were more frequently detected, high-grade tumors were not associated with AF1q expression in dependence of the Perzin/Szanto grading system (*p* = 0.70). Interestingly, patients with more than 50% of solid tumor tissue were only detected in the AF1q^high^ group and strictly solid tumors were just found within this group as well. However, statistical significance was missed (*p* = 0.0502). Perineural invasion was equally distributed in the AF1q^low^ and AF1q^high^ tumor group (*p >* 0.99).

**Table 2:** Comparison and statistical analysis of AF1q^low^ and AF1q^high^ patients

	AF1q^low^	AF1q^high^	p value
Total number of patients	22	24	
T-classification			0.9368
T1	3	3	
T2	4	3	
T3	4	4	
T4	11	14	
Lymph node metastases	2	8	0.0740
Distant metastases (initially)	1	2	0.9999
Distant recurrences	8	10	0.7693
Perineural Invasion	11	12	0.9999
Solid components	3	10	0.0509
High Grad Malignancy			
Perzin/Szanto	3	5	0.7021
Spiro	0	5	0.0502
Localized in Minor Salivary Glands	15	15	0.7628
Death	6	19	0.0009
Recurrence	10	13	0.7683

**Fig. 2:**
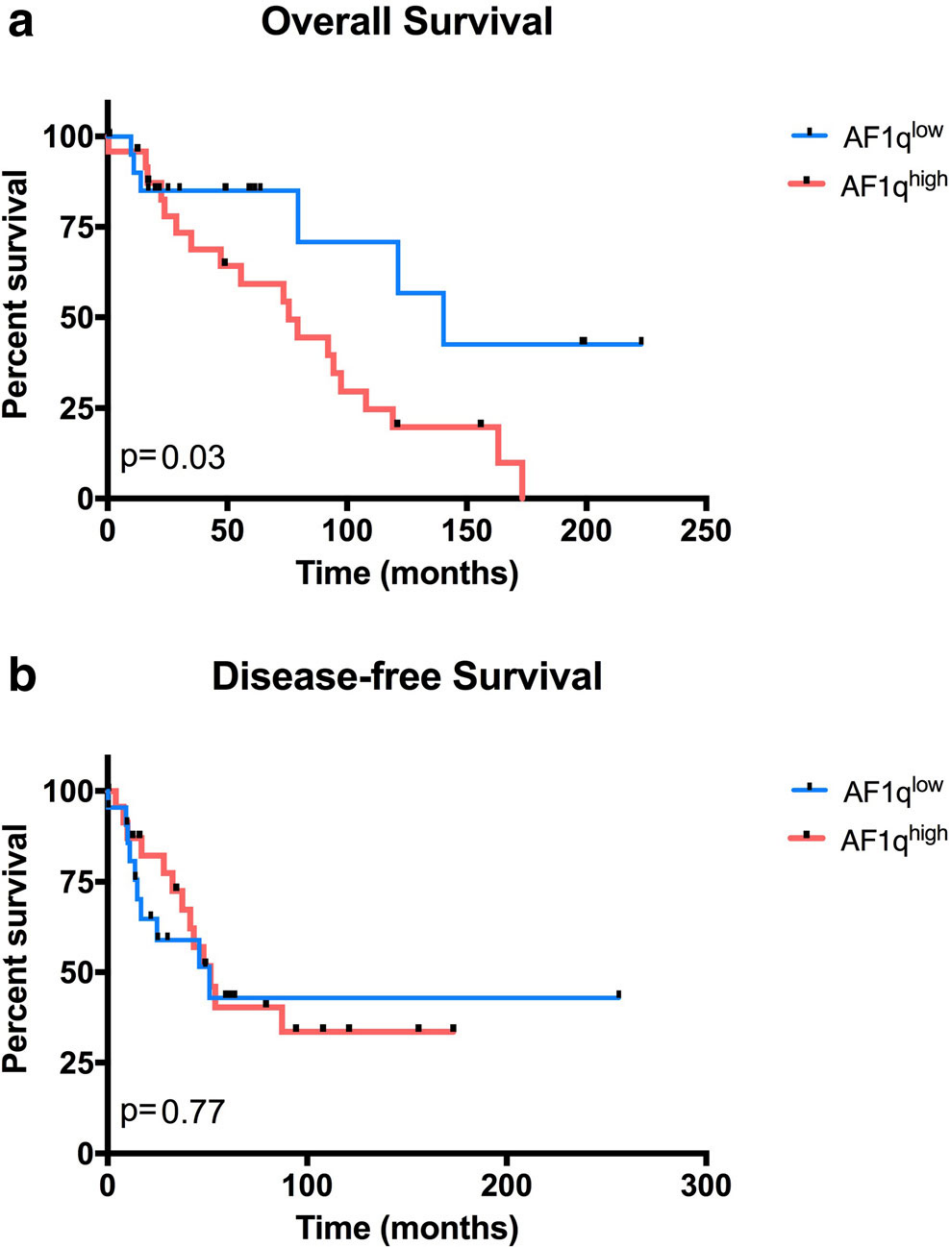
Patients were stratified into AF1q^low^ and AF1q^high^. Overall survival in dependence of AF1q expression is shown in (**a**). Median survival in AF1q^low^ measured 140.3 months and 75.7 months in AF1q^high^ (*p* = 0.03). Disease free survival (**b**) showed no significant difference in outcome (*p* = 0.77)

Survival analysis revealed a mortality rate almost three times higher in patients with AF1q^high^ tumors (79.1% vs. 27.3%, *p* = 0.001), whereas the recurrence rate was not significant in patients with AF1q^high^ tumors (54.2% vs. 45.4%, *p* = 0.77). Kaplan-Meier curves showed a median OS in patients with AF1q^low^ tumors (140.3 months) and in patients with AF1q^high^ tumors (75.7 months). Log-rank tests showed that high expression of AF1q significantly worsened OS in ACC patients (*p* = 0.03). Multivariate analysis revealed a significant difference on distant metastases and T1 vs. T4 tumors (data not shown). Analysis of DFS indicated no significant difference between both groups (AF1q^high^ 51.6 months vs. AF1q^low^ 51.1 months, *p* = 0.77). (Fig. 2a, b).

### AF1q Expression Is Associated with Up-Regulation of CD44 and STAT3

Previous studies showed an association of AF1q expression with expression of STAT3 and the Wnt downstream target CD44.

**Fig. 3:**
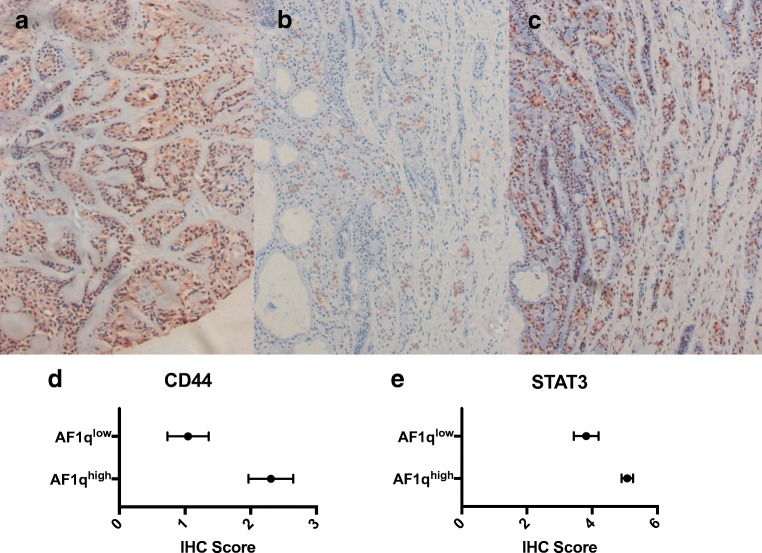
Examples of AF1q (**a**), CD44 (**b**) and STAT3 (**c**) expression patterns observed in the same patient. Additionally, AF1q^high^ patients showed significant higher co-expression of CD44 (D) and STAT3 (**e**)

The group of patients with AF1q^high^ tumors included 9 patients (34.6%) with a STAT3 score of 6 and 12 patients (46.2%) with a STAT3 score of 5. (Fig. 3). In contrast, only 2 patients (9.1%) in the AF1q^low^ group had a STAT3 score of 6 and 9 patients (40.9%) a score of 5. Mann-Whitney tests revealed that STAT3 was significantly higher expressed in AF1q^high^ patients (*p* = 0.002). Next, ROC curves were computed, revealing a moderate correlation of STAT3 with AF1q expression (Spearman r correlation coefficient = 0.53).

CD44 showed lower expression levels than STAT3 in our cohort. Only 2 patients (7.7%) expressed CD44 with a score of 6 in the AF1q^high^ group, and no patient with a score of 6 was detected in the AF1q^low^ group. No expression of CD44 was found in 6 patients (23.1%) with AF1q^high^ expression levels and in 13 patients (59.1%) with AF1q^low^ expression. Again, Mann-Whitney test showed that AF1q^high^ patients had a significantly higher expression of CD44, with a moderate correlation being observed between AF1q and CD44 expression (Spearman r correlation coefficient = 0.44).

## Discussion

In our study expression of AF1q was immunohistochemically detectable in almost two thirds of the patients, and more than half of our cohort expressed AF1q at a high or moderate level. Expression of AF1q was found in a number of other solid malignancies such as breast cancer, ovarian cancer, and colorectal cancer. [7, 19, 20] Subsequently, we compared survival of patients with AF1q^high^ and AF1q^low^ tumors in our study. One of the major findings was that patients with AF1q^high^ tumors showed a significant decrease in OS. Other studies reported similar findings. Hu and colleagues were able to demonstrate via The Cancer Genome Atlas dataset of colorectal cancer patients that AF1q expression resulted in reduced OS and DFS rates. Park and coworkers reported similar results in breast cancer patients. [7] In addition, their study group was able to identify AF1q as a prognosticator for brain metastases. Interestingly, we did not find any difference in the occurrence of distant metastases at the time of initial diagnosis or in the course of disease. Nevertheless, lymph node metastases were detected >3 times more often in patients with AF1q^high^ tumors. However the level of significance was not reached. The fact of AF1q not being associated with metastatic disease might be contributed to the low number of patients eligible for this analysis. In a study investigating 96 colorectal cancer patients, AF1q upregulation was significantly associated with lymph node metastases [19] Moreover, expression of AF1q was predominately found in patients with solid components of ACC. A solid growth pattern in ACCs is generally associated with worse clinical outcome. Strictly solid ACCs were only found in AF1q^high^ patients.

In addition, we examined the expression of CD44, as a downstream target of the Wnt pathway, and of STAT3 in our cohort. Our data suggests that AF1q expression may lead to an activation of both pathways since CD44 and STAT3 are expressed at a higher intensity in AF1q^high^ patients. The promoting role of AF1q for the oncogenic STAT3 pathway was described by Park et al. [14] Furthermore, AF1q has been shown to induce expression of PDGF-B/PDGFBR-B, which results in a markedly enhanced STAT3 activity. Additionally, AF1q results in the transcriptional activation of CD44 downstream of Wnt. [7] Activated Wnt signaling has recently been associated with AF1q activation and the enhancement of cancer stem cell population in breast cancer. [21]

In conclusion, we have been able to link the expression of AF1q in patients with ACC to a significant decrease in OS. Our results could not confirm higher rates of distant metastases in ACC patients compared to other data reported in the literature. However, lymph node metastases were more often found in patients AF1q^high^ tumors. Additionaly, we linked AF1q expression to higher levels of STAT3 and CD44. As a matter of fact, association with the STAT3 as well as the Wnt signaling pathway might open new therapeutic perspectives in fighting ACC. Taken together, our results argue in favor of further investigation of AF1q in ACC of the salivary glands in order to confirm our results and to examine AF1q as a potential therapeutic target.
